# Supplementary material for: Identification of oleoylethanolamide as an endogenous ligand for HIF-3α
Source: Nat Commun. 2022 May 9;13:2529. doi: 10.1038/s41467-022-30338-z (PMC9085743; doi:10.1038/s41467-022-30338-z)
Supplement: Supplementary file 1 — Supplementary Information [file 41467_2022_30338_MOESM1_ESM.pdf]

Supplementary Table 1. Data collection and refinement statistics

	HIF-3α-ARNT	HIF-3α-ARNT-OEA
**PDB ID**	7V7L	7V7W
**Data collection**		
Space group	P 212121	P 212121
Cell dimensions		
a, *b*, *c* (Å)	65.84, 86.29, 143.70	65.94, 86.79, 143.72
α, β, γ (°)	90, 90, 90	90, 90, 90
Resolution (Å)	50.0-2.30 (2.34-2.30) *	50.0-2.50 (2.54-2.50)
R_{merge}	6.7 (109.6)	9.1 (123.1)
$I / \sigma I$	39.6 (2.0)	26.1 (2.0)
Completeness (%)	99.9 (98.7)	99.9 (100.0)
Redundancy	13.0 (11.8)	10.8 (10.8)
**Refinement**		
Resolution (Å)	36.99-2.30 (2.38-2.30)	35.93-2.51 (2.60-2.51)
No. reflections	36472 (3091)	27947 (2028)
R_{work} / R_{free}	20.4/24.0 (29.8/37.1)	21.7/25.6 (27.6/29.4)
No. atoms		
Protein	4611	4521
Ligand/ion	-	23
Water	266	149
B-factors		
Protein	46.7	50.5
Ligand/ion	-	51.0
Water	42.9	47.4
R.m.s. deviations		
Bond lengths (Å)	0.007	0.004
Bond angles (°)	1.17	1.15

One crystal was used for each structure.

*Values in parentheses are for highest-resolution shell.

Supplementary Table 2. Comparison of the per-residue decomposition of the MM-GBSA $\Delta G_{binding}$ between the *apo* and holo HIF-3α-ARNT dimers at the HIF-3α PAS-B-ARNT A/B loop interface.

ARNT		
Residue	$HIF3^{noOEA}$	$HIF3^{OEA}$
	$\Delta G_{binding}$ (kcal/mol)	
P349	-2.01304	-4.294163
N350	0.432213	-3.576467
C351	-4.76865	-0.788034
T352	-0.61774	-1.633032
D353	0.309226	-3.235826
M354	-2.44126	-2.747138
S355	0.104693	0.2845694
N356	0.397567	0.1703358
I357	-1.47821	-0.068817
C358	-0.2969	0.0297909
Q359	0.169755	0.0670594
P360	-0.0139	0.0377723

HIF-3α		
Residue	$HIF3^{noOEA}$	$HIF3^{OEA}$
	$\Delta G_{binding}$ (kcal/mol)	
A278	-3.40279	-3.137239
L279	-2.7605	-3.915723
D280	0.97584	0.3716415
S281	-1.77174	-1.298974
D282	0.252466	0.4640641
A283	0.027287	0.0745248
V284	-0.16465	-0.128109
S285	-0.37742	-0.096779
R286	0.272011	0.1967184
S287	0.185877	0.1199604
I288	-0.64223	-0.71874
H289	-0.84072	-0.683273
T290	0.168426	0.1520495
L291	-0.0084	-0.026515
L292	-1.65198	-1.613201
S293	0.111257	0.1276733
K294	0.310891	0.4296634
G295	0.027525	0.0263762
Q296	0.246079	0.2196832
A297	0.091327	0.0760297
V298	0.025524	-0.181759
T299	0.375249	-0.020427
G300	-1.46353	-0.348903
Q301	-0.59506	0.7670256
Y302	-0.40938	-0.292653
R303	-0.09955	-5.561327
F304	-0.13919	-0.055267
L305	-0.35102	-0.5344

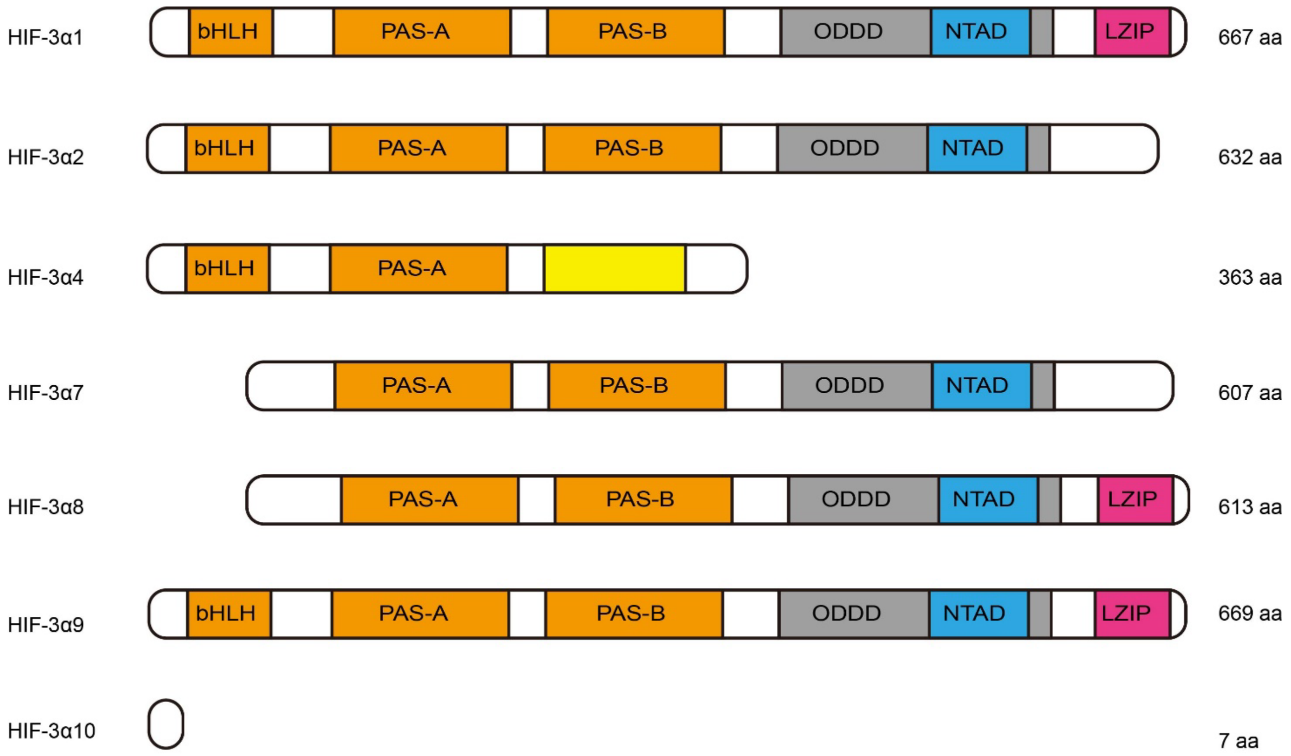

Supplementary Fig. 1 | Multiple splice variants of human HIF-3α protein. The variants of HIF-3α confirmed in human cells are HIF-3α1, HIF-3α2, HIF-3α4, HIF-3α7, HIF-3α8, HIF-3α9, and HIF-3α10. The protein domain organizations and the numbers of amino acids of these variants are listed above. bHLH, basic helix-loop-helix; PAS, PER-ARNT-SIM; ODDD, oxygen-dependent degradation domain; NTAD, N-terminal transactivation domain; LZIP, leucine zipper.

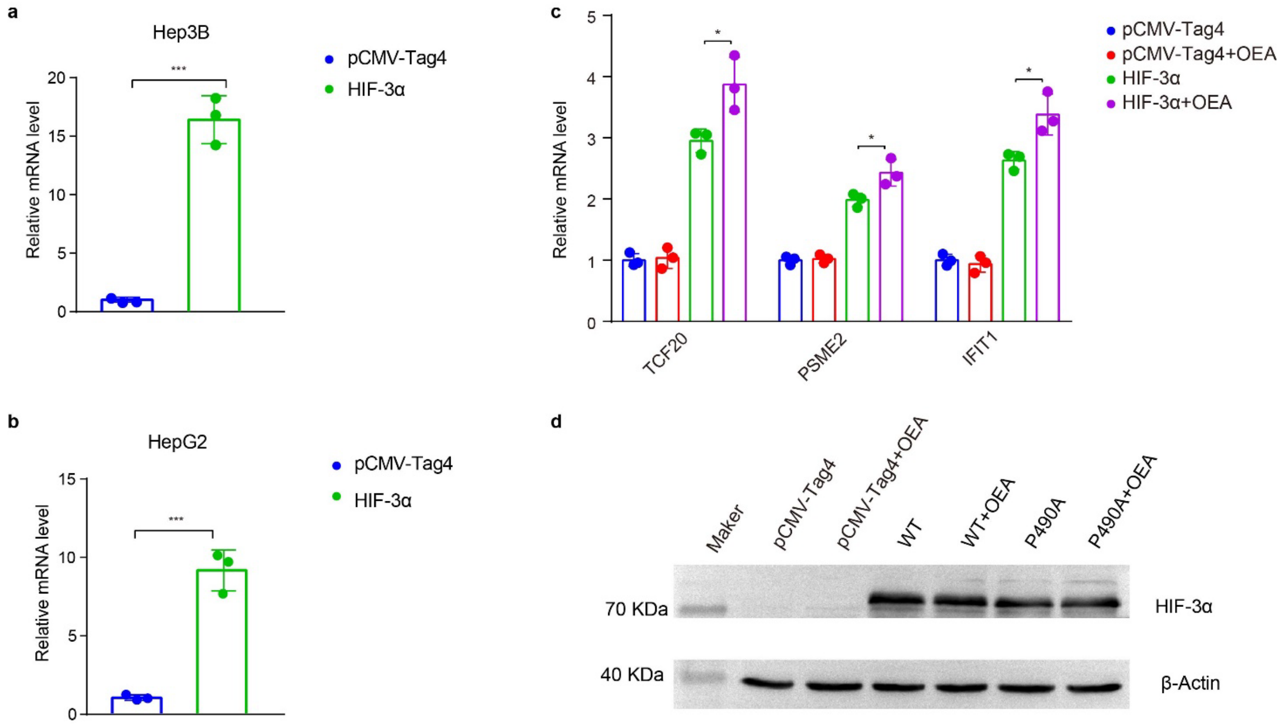

Supplementary Fig. 2 | HIF-3α overexpression elevates the transcription level of potential target genes. a,b, The expression of *HSPA6* were up-regulated by HIF-3α1 overexpression in Hep3B (**a**) and HepG2 (**b**) cells. *** $p = 0.0002$ for pCMV-Tag4 vs. HIF-3α in Hep3B cells, *** $p = 0.0004$ for pCMV-Tag4 vs. HIF-3α in HepG2 cells. **c,** The effects of HIF-3α1 overexpression and 25 μM OEA treatment on the mRNA levels of *TCF20*, *PSME2*, *IFIT1* in HEK293 cells. * $p = 0.0308$ for HIF-3α vs. HIF-3α+OEA on *TCF20*, * $p = 0.0344$ for HIF-3α vs. HIF-3α+OEA on *PSME2*, * $p = 0.0234$ for HIF-3α vs. HIF-3α+OEA on *IFIT1*. **d,** HIF-3α1 and its degradation-resistant P490A mutant were overexpressed under normal oxygen in HEK293 cells treated with or without OEA (25 μM), and the empty vector pCMV-Tag4 was used as a control. Western blotting was conducted to detect the intrinsic, overexpressed wild-type (WT) or P490A mutant proteins. The experiment was repeated three times, and data for one representative are shown. Error bars, mean ± SD.; n=3 (distinct samples for cell-based assays); statistical significance: * $p < 0.05$, *** $p < 0.001$ (Unpaired two-tailed t test).

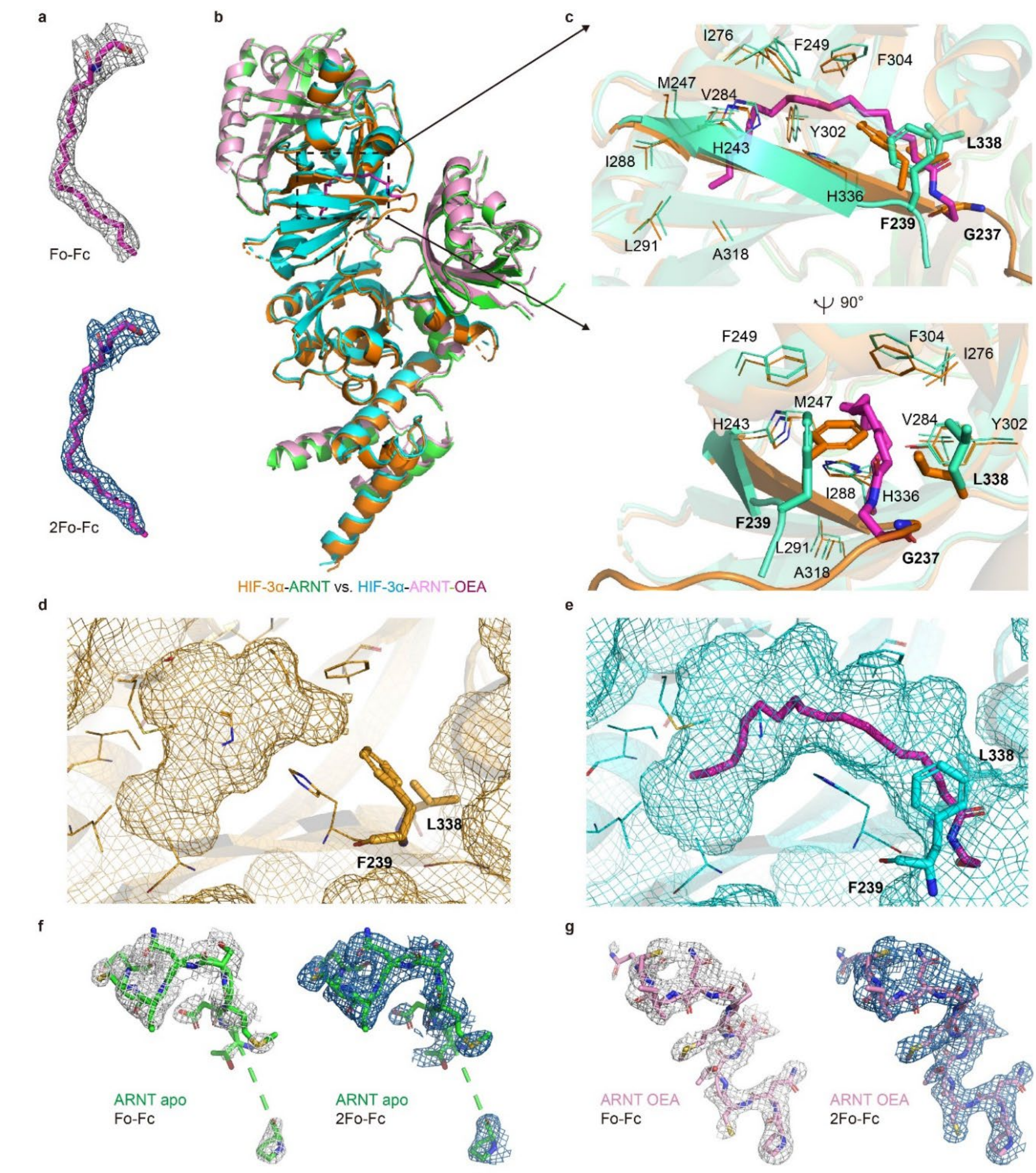

Supplementary Fig. 3 | Crystal structure of HIF-3α-ARNT in complex with OEA. a, The Fo-Fc omit map (contour level = 3.0 σ, shown as the grey mesh) and the 2Fo-Fc map (contour level = 0.8 σ, shown as the blue mesh) of OEA in the HIF-3α-ARNT complex structure. **b,** Superimposition of HIF-3α-ARNT structures in the "*apo*" form and in complex with OEA. HIF-3α and ARNT proteins in complex with OEA are colored in cyan and pink; whereas in the "*apo*" form they are colored in orange and green, respectively. **c,** An enlarged view of OEA binding pocket in the superimposed two forms. Residues with no obvious conformational changes in the two forms are shown as lines; and the three residues G237, F239 and L338 with clear changes are shown as sticks. **d,e,** The HIF-3α PAS-B pockets in "*apo*" form (**d**) and in complex with OEA (**e**) are both shown in mesh. Two key residues (F239 and L338) gating the inner cavity are highlighted in sticks. **f,g** The Fo-Fc omit map (contour level = 3.0 σ, shown as the grey mesh) and the 2Fo-Fc map (contour level = 1.0 σ, shown as the blue mesh) of ARNT A/B loop in the *apo* structure (green) and in the OEA-bound structure (pink).

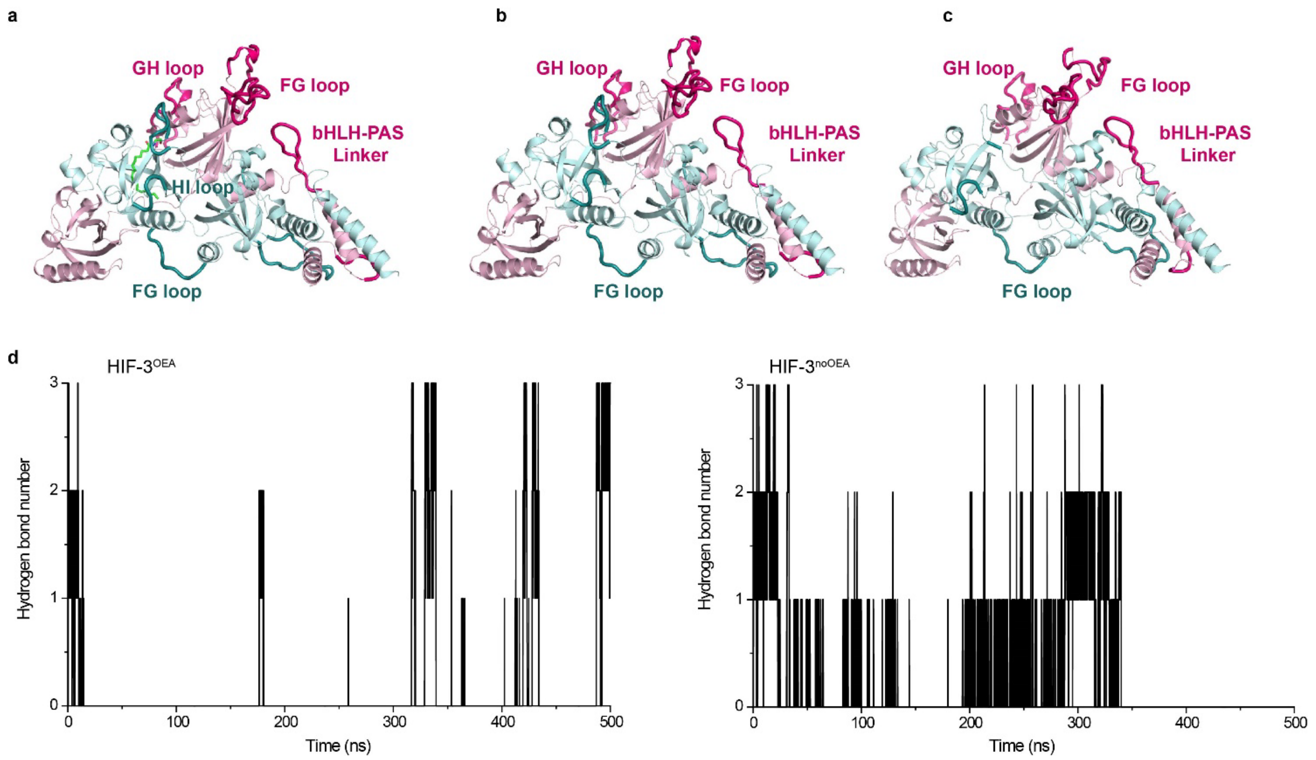

Supplementary Fig. 4 | The molecular dynamics (MD) simulations on three systems. a-c, Cartoon representation of the HIF-3^{OEA} (**a**), HIF-3^{noOEA} (**b**) and HIF-3^{apo} (**c**) structures with the modelled linkers: ARNT in pink, HIF-3α in cyan, and OEA in green. Modelled segments are shown in darker colors. **d,** Hydrogen bonds between the ARNT A/B loop and HIF-3α PAS-B domain during MD simulation of HIF-3^{OEA} and HIF-3^{noOEA} systems, respectively.

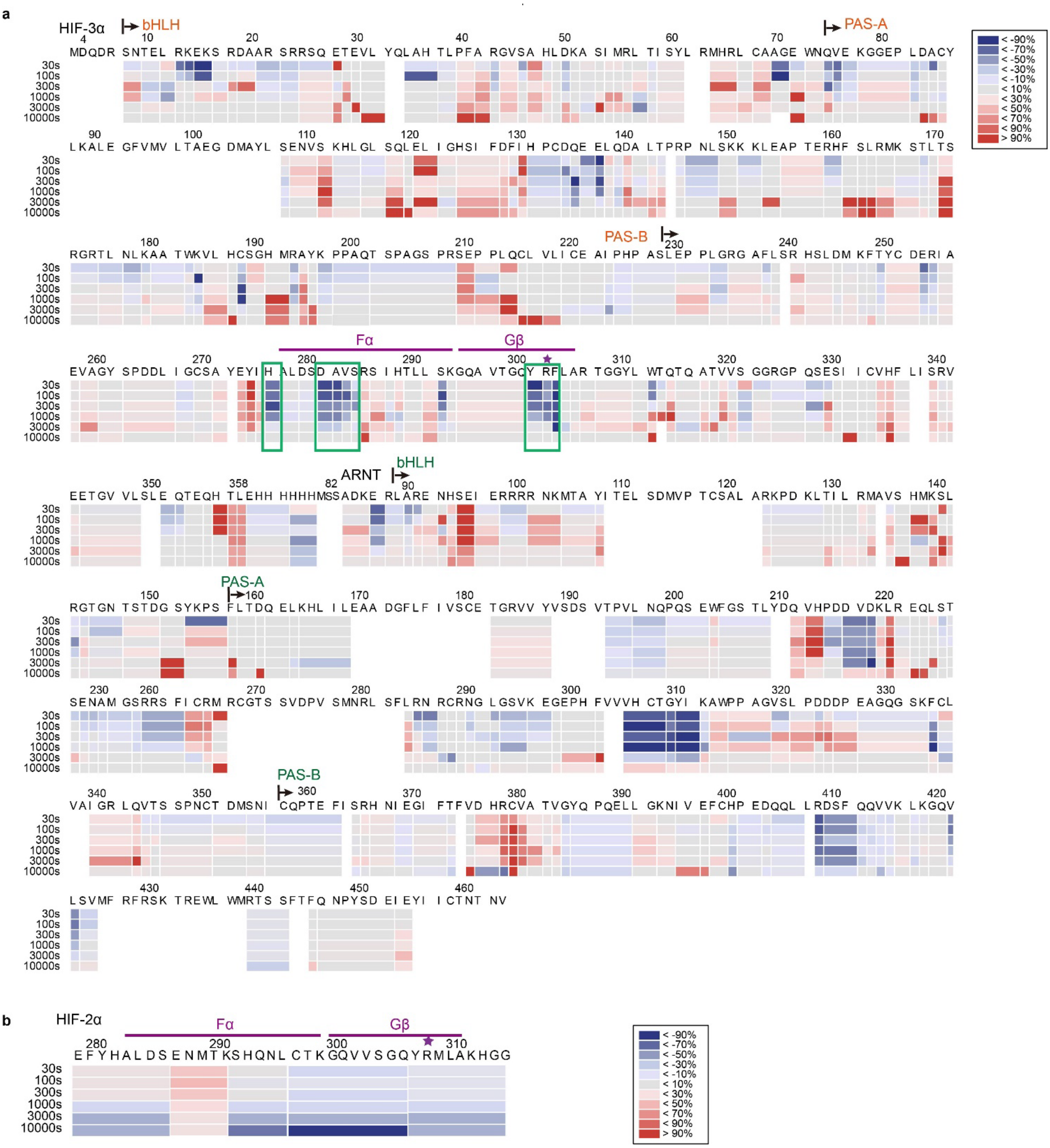

Supplementary Fig. 5 | Agonist binding enhances the interactions between HIF-α and ARNT as shown in the HDX-MS patterns. a, Differences in the deuteration level of HIF-3α and ARNT (OEA-bound minus "*apo*" state) at various time points (30, 100, 300, 1000, 3000 and 10,000 s). Fα and Gβ of HIF-3α are labeled above the sequence, and the key residues stabilized are boxed. **b,** Differences in the deuteration level of HIF-2α Fα and Gβ regions, induced by the agonist M1001 at various time points (30, 100, 300, 1000, 3000 and 10,000 s). R303 and R308 are marked with asterisks.

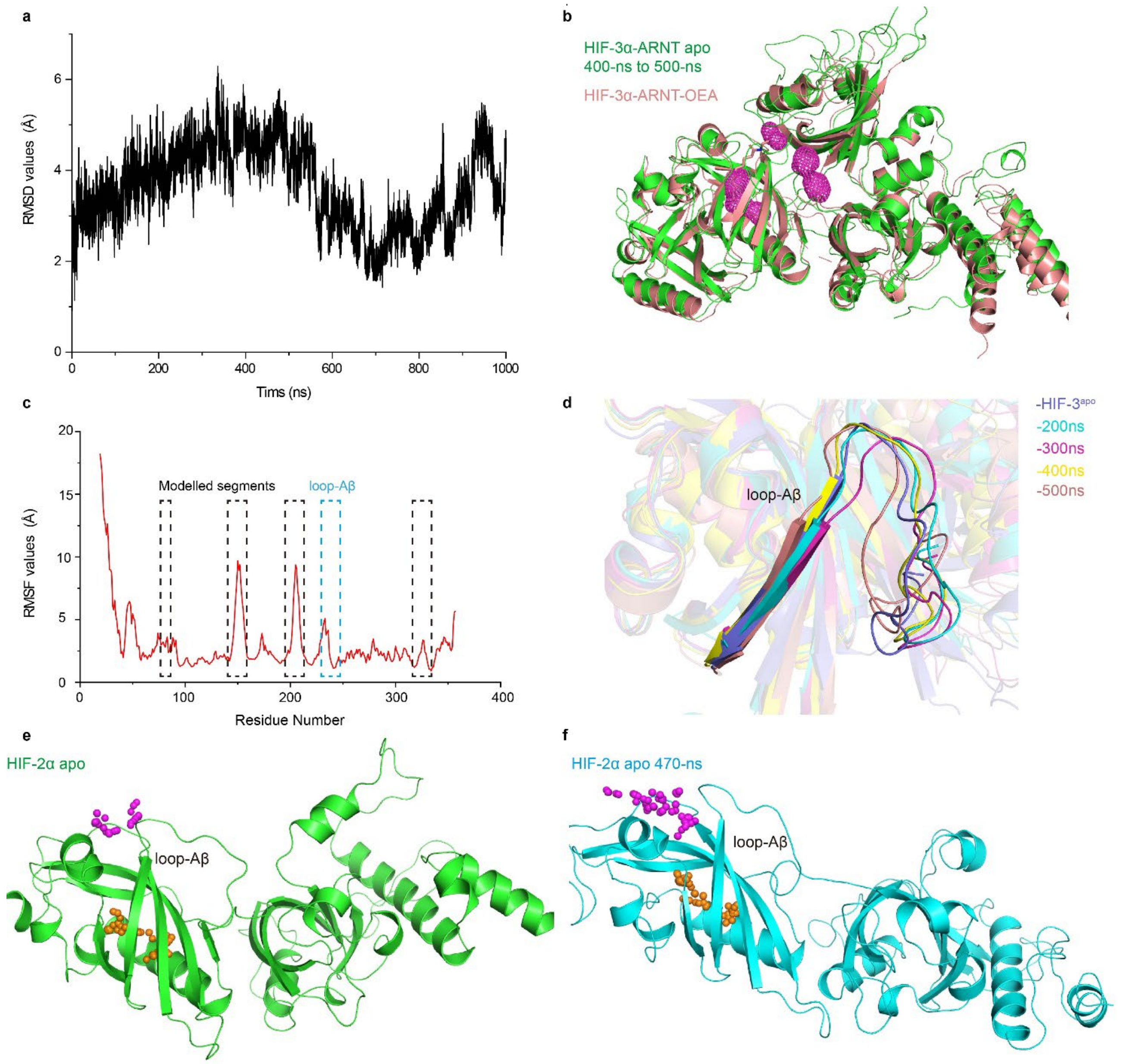

Supplementary Fig. 6 | The loop-Aβ region gating the PAS-B entrance is flexible in HIF-α. a, Cα RMSD plots for loop-Aβ (residues 227-244) in the HIF-3^{apo} system. **b**, Position of the pocket detected during the MD trajectory of HIF-3^{apo} from 400-ns to 500-ns. **c**, Cα RMSF plots for the HIF-3α in monomeric system. The highly flexible regions including modelled linkers (black) and loop-Aβ (cyan) are highlighted. **d,** Snapshot structures extracted from the trajectories of monomeric HIF-3^{apo}. **e,f,** Two binding pockets predicted by Fpocket for the initial structure (**e**) and the 470-ns snapshot (**f**) of HIF-2α are shown as spheres. The orange sphere region is the ligand binding pocket within the PAS-B domain, and the magenta one is the entry pocket.

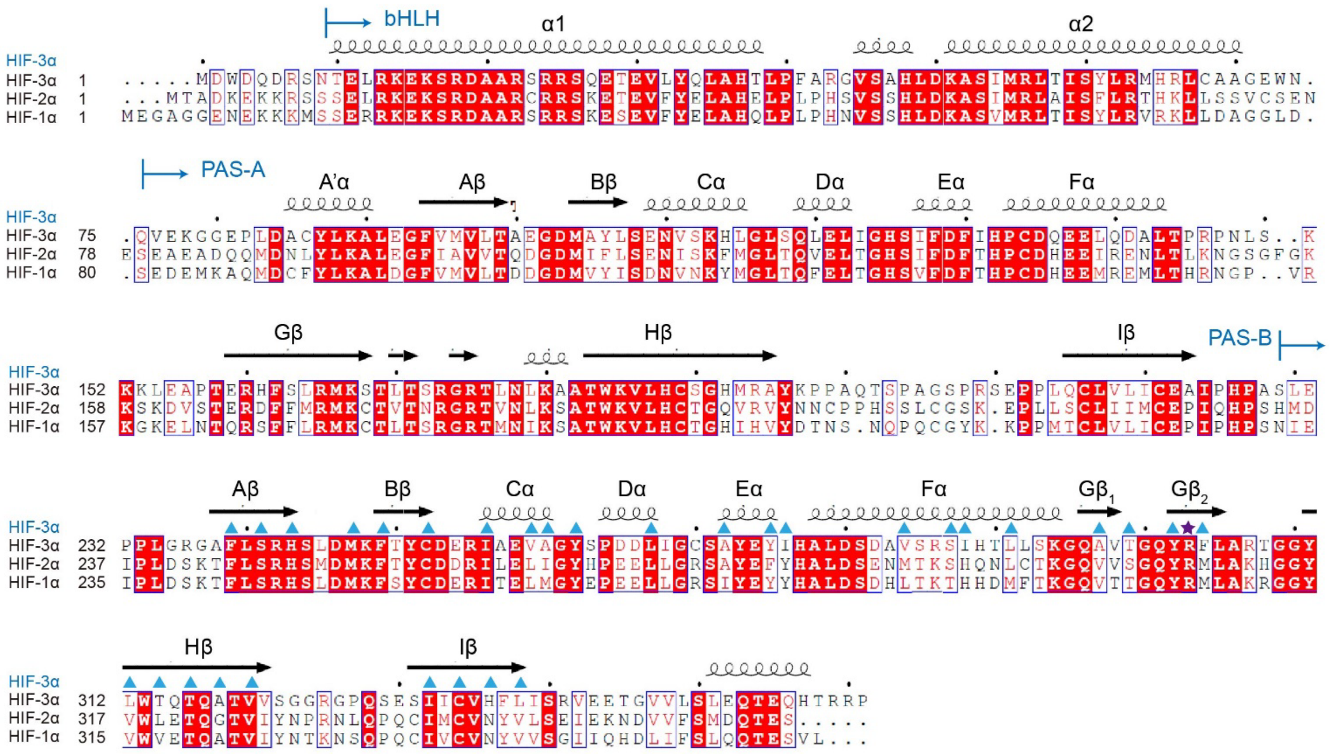

Supplementary Fig. 7 | Structure-based sequence alignment of mouse HIF-1α, HIF-2α, HIF-3α proteins. The domain ranges and secondary structures are labeled above the sequence. HIF-3α R303 is marked with a purple asterisk, and the residues outlining the PAS-B pocket are highlighted by blue triangles.

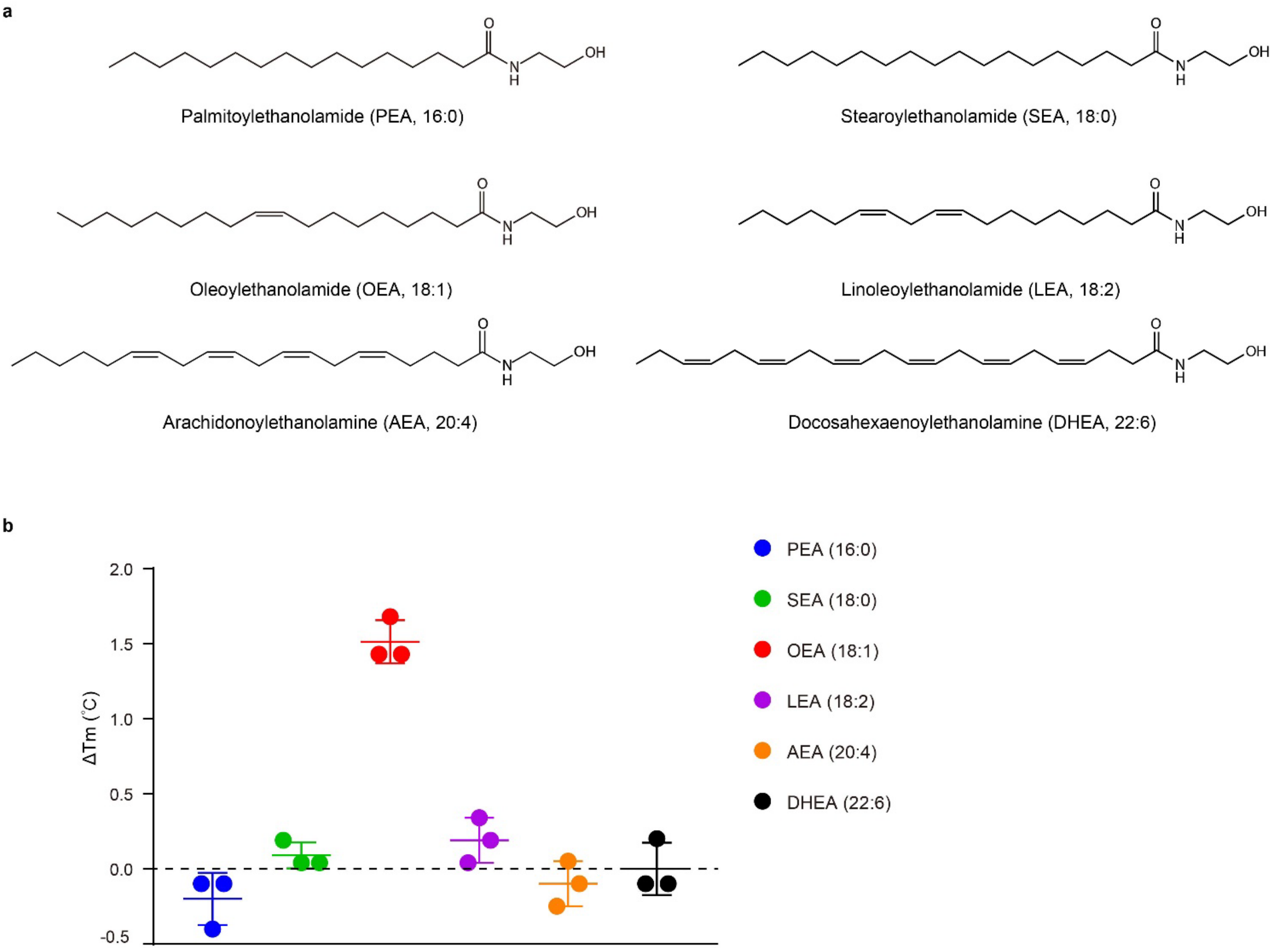

Supplementary Fig. 8 | HIF-3α prefers to bind OEA among a number of related NAEs. a, Chemical structures of the selected *N*-acylethanolamines (NAEs). The numbers of carbon atoms in the lipid chains and unsaturated bonds are labelled separately. **b,** The changes in protein melting temperatures (ΔT_{m}) of the HIF-3α-ARNT complex in the presence of NAEs measured by thermal shift assays. Error bars, mean ± SD.; $n = 3$ (biological replicates).

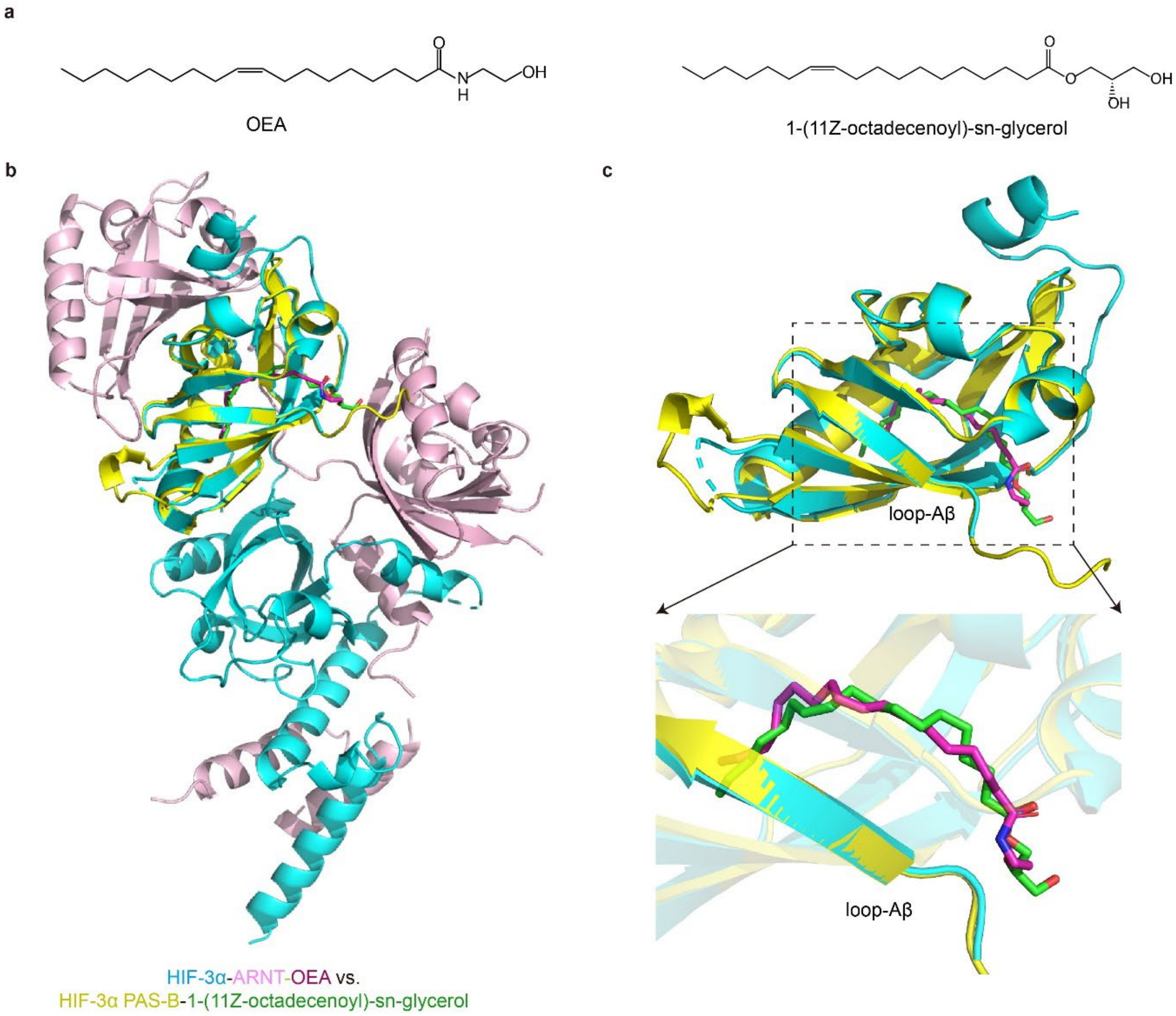

Supplementary Fig. 9 | The comparison of HIF-3α-ARNT-OEA and HIF-3α PAS-B-1-(11Z-octadecenoyl)-sn-glycerol crystal structures. a, Chemical structures of OEA and 1-(11Z-octadecenoyl)-sn-glycerol. **b,** Superimposition of multi-domain HIF-3α-ARNT in complex with OEA and the single HIF-3α PAS-B in complex with 1-(11Z-octadecenoyl)-sn-glycerol[27] (PDB code: 4WN5). **c,** Comparison of OEA and 1-(11Z-octadecenoyl)-sn-glycerol both bound within the HIF-3α PAS-B domain. HIF-3α and ARNT in the heterodimer are colored in cyan and pink, respectively; while the single HIF-3α PAS-B domain is colored in yellow. OEA and 1-(11Z-octadecenoyl)-sn-glycerol are shown in magenta and green, respectively.
