# Supplementary material for: Identification of oleoylethanolamide as an endogenous ligand for HIF-3α
Source: Nat Commun. 2022 May 9;13:2529. doi: 10.1038/s41467-022-30338-z (PMC9085743; doi:10.1038/s41467-022-30338-z)
Supplement: Supplementary file 3 — Reporting Summary [file 41467_2022_30338_MOESM3_ESM.pdf]

nature portfolio

Corresponding author(s): Dalei Wu

Last updated by author(s): Apr 25,2022

Reporting Summary

Nature Portfolio wishes to improve the reproducibility of the work that we publish. This form provides structure for consistency and transparency in reporting. For further information on Nature Portfolio policies, see our Editorial Policies and the Editorial Policy Checklist.

Statistics

For all statistical analyses, confirm that the following items are present in the figure legend, table legend, main text, or Methods section.

n/a	Confirmed	
☐	☒	The exact sample size (*n*) for each experimental group/condition, given as a discrete number and unit of measurement
☐	☒	A statement on whether measurements were taken from distinct samples or whether the same sample was measured repeatedly
☐	☒	The statistical test(s) used AND whether they are one- or two-sided<br>*Only common tests should be described solely by name; describe more complex techniques in the Methods section.*
☒	☐	A description of all covariates tested
☒	☐	A description of any assumptions or corrections, such as tests of normality and adjustment for multiple comparisons
☐	☒	A full description of the statistical parameters including central tendency (e.g. means) or other basic estimates (e.g. regression coefficient) AND variation (e.g. standard deviation) or associated estimates of uncertainty (e.g. confidence intervals)
☐	☒	For null hypothesis testing, the test statistic (e.g. *F*, *t*, *r*) with confidence intervals, effect sizes, degrees of freedom and *P* value noted<br>*Give P values as exact values whenever suitable.*
☒	☐	For Bayesian analysis, information on the choice of priors and Markov chain Monte Carlo settings
☒	☐	For hierarchical and complex designs, identification of the appropriate level for tests and full reporting of outcomes
☒	☐	Estimates of effect sizes (e.g. Cohen's *d*, Pearson's *r*), indicating how they were calculated

Our web collection on statistics for biologists contains articles on many of the points above.

Software and code

Policy information about availability of computer code

Data collection	Crystal data were collected on beamlines BL19U1 at the Shanghai Synchrotron Radiation Facility (SSRF); Protein thermal shift assay was performed using a QuantStudio 3 (Applied Biosystems); SPR data were collected on a Biacore T200 (GE Healthcare) ; TR-FRET data were collected on Spark microplate reader (Tecan); RT-PCR data were collected on a LightCycler 480 system (Roche); HDX-MS data were collected from Thermo LTQ Orbitrap-Elite mass spectrometer with a Thermo H-ESI II probe; MD simulations data were collected using Gromacs 2019.6 program package and AMBER 16 software package.
Data analysis	HKL3000 (v716.1); Coot (v0.8.9); Phenix (v1.14-3260); PyMOL (v2.3); ESPript(3.x); Protein Thermal Shift™ Software (v1.30); BIA evaluation (v3.0.2); Gromacs (v2019.6); AMBER (v16); HDExaminer (v2.0); GraphPad Prism (v7.0); Fpocket (v4.0); PyVOL (v1.7.6).

For manuscripts utilizing custom algorithms or software that are central to the research but not yet described in published literature, software must be made available to editors and reviewers. We strongly encourage code deposition in a community repository (e.g. GitHub). See the Nature Portfolio guidelines for submitting code & software for further information.

Data

Policy information about availability of data

All manuscripts must include a data availability statement. This statement should provide the following information, where applicable:

- Accession codes, unique identifiers, or web links for publicly available datasets
- A description of any restrictions on data availability
- For clinical datasets or third party data, please ensure that the statement adheres to our policy

The data that support the findings of this study are available within the paper and its Supplementary Information files. Coordinates and structure factors of the

HIF-3α-ARNT protein complexes in "apo" and OEA-bound forms, have been deposited in the Protein Data Bank under accession codes 7V7L [http://doi.org/10.2210/pdb7V7L/pdb] and 7V7W [http://doi.org/10.2210/pdb7V7W/pdb], respectively. The accession codes for HIF-2α-ARNT, HIF-2α-ARNT-PT2385, HIF-2α-ARNT-M1001 and HIF-3α PAS-B-1-(11Z-octadecenoyl)-sn-glycerol are 4ZP4 [http://doi.org/10.2210/pdb4ZP4/pdb], 6E3S [http://doi.org/10.2210/pdb6E3S/pdb], 6E3U [http://doi.org/10.2210/pdb6E3U/pdb] and 4WN5 [http://doi.org/10.2210/pdb4WN5/pdb], respectively. The HDX-MS data obtained in this study are available in the PRIDE database under accession code PXD033376 [http://proteomecentral.proteomexchange.org/cgi/GetDataset?ID=PXD033376]. Source data are also provided.

Field-specific reporting

Please select the one below that is the best fit for your research. If you are not sure, read the appropriate sections before making your selection.

☒ Life sciences ☐ Behavioural & social sciences ☐ Ecological, evolutionary & environmental sciences

For a reference copy of the document with all sections, see nature.com/documents/nr-reporting-summary-flat.pdf

Life sciences study design

All studies must disclose on these points even when the disclosure is negative.

Sample size	Experiments were performed three times independently unless indicated. Choice of sample sizes guided by established precedents from leading works in the field. [see for example Wu et al. Nat Chem Biol. 2019 Apr;15(4):367-376]
Data exclusions	No data were excluded.
Replication	All experiments were repeated at least twice with similar results. The number of biological replicates is stated in the figure legends.
Randomization	No experimental grouping requiring randomization was performed. The positions of samples on multi-well plates were different among individual experiments to minimize the systematic errors.
Blinding	Not applicable, as no animal or clinical experiments were involved and data were automatic readouts of the instruments.

Reporting for specific materials, systems and methods

We require information from authors about some types of materials, experimental systems and methods used in many studies. Here, indicate whether each material, system or method listed is relevant to your study. If you are not sure if a list item applies to your research, read the appropriate section before selecting a response.

Materials & experimental systems

n/a	Involved in the study	
☐	☒	Antibodies
☐	☒	Eukaryotic cell lines
☒	☐	Palaeontology and archaeology
☒	☐	Animals and other organisms
☒	☐	Human research participants
☒	☐	Clinical data
☒	☐	Dual use research of concern

Methods

n/a	Involved in the study	
☒	☐	ChIP-seq
☒	☐	Flow cytometry
☒	☐	MRI-based neuroimaging

Antibodies

Antibodies used	The primary antibodies HIF3A Polyclonal Antibody (Proteintech, 27650-1-AP) and Beta Actin Monoclonal Antibody (Proteintech, 66009-1-Ig, Clone No. 2D4H5) were used as 1:1000 dilution in the western blotting; and the secondary antibodies Goat Anti-Rabbit IgG (Sangon Biotech, D110058) and HRP-conjugated Goat Anti-Mouse IgG (Sangon Biotech, D110087) were used as 1:6000 dilution in the western blotting.Mab Anti-6HIS-Tb cryptate Gold (Cisbio 61H12TLF) used at 1.05 ng per well in the TR-FRET-based binding assay.
Validation	Antibodies were validated for the indicated use by the manufacturer available on their websites:<br>1. HIF3A Polyclonal Antibody<br>https://www.ptglab.com/Products/HIF3A-Antibody-27650-1-AP.htm<br>2. Beta Actin Monoclonal Antibody<br>https://www.ptglab.com/products/Pan-Actin-Antibody-66009-1-Ig.htm<br>3. Goat Anti-Rabbit IgG<br>https://www.sangon.com/productDetail?productInfo.code=D110058<br>4. HRP-conjugated Goat Anti-Mouse IgG<br>https://www.sangon.com/productDetail?productInfo.code=D110087<br>5. Mab Anti-6HIS-Tb cryptate Gold<br>https://www.cisbio.cn/mab-anti-6his-tb-cryptate-gold-40041#:~:text=MAb%20Anti-6HIS-Tb%20cryptate%20Gold%20is%20a%20monoclonal%20antibody,It%20recognizes%20synthetic%20polyhistidine%20or%20polyhistidine-tagged%20fusion%20protein.

Eukaryotic cell lines

Policy information about cell lines

Cell line source(s)	HEK293 cells (Procell CL-0001) ; Hep3B (Beijing Dingguo CS0172) ; HepG2 (Procell CL-0103)
Authentication	Cell authentication was performed using the short tandem repeats (STR) method by the respective vendors.
Mycoplasma contamination	These cell lines were all tested negative for mycoplasma contamination.
Commonly misidentified lines (See ICLAC register)	No commonly misidentified cell lines were used in this study.
